# Combination of Gluten-Digesting Enzymes Improved Symptoms of Non-Celiac Gluten Sensitivity: A Randomized Single-blind, Placebo-controlled Crossover Study

**DOI:** 10.1038/s41424-018-0052-1

**Published:** 2018-09-19

**Authors:** Hiroki Ido, Hirotaka Matsubara, Manabu Kuroda, Akiko Takahashi, Yuzo Kojima, Satoshi Koikeda, Makoto Sasaki

**Affiliations:** 1 Amano Enzyme Inc., Nagoya, Japan; 2 Department of Gastroenterology, Aichi Medical University School of Medicine, Nagakute, Japan

## Abstract

**Introduction:**

Recently, the population of individuals with non-celiac gluten sensitivity (NCGS) who do not have celiac disease but show improved symptoms with a gluten-free diet, has increased. Enzyme replacement therapy using digestive enzymes is expected to improve the symptoms of NCGS and be sustainable, since gluten-related proteins that are indigestible by the digestive system have been considered triggers of NCGS.

**Methods:**

We selected patients with NCGS by screening demographic interviews, as well as performing medical evaluations, anti-gluten antibody tests, and gluten challenge tests. We performed a single-blind and crossover clinical trial with these subjects using a gluten challenge with the enzyme mixture or a placebo. Our designed enzyme mixture contained peptidase, semi alkaline protease, deuterolysin, and cysteine protease derived from *Aspergillus oryzae*, *Aspergillus melleus*, *Penicillium citrinum*, and *Carica papaya* L., respectively.

**Results:**

Administration of the enzyme mixture significantly decreased the change in the score of the symptom questionnaire before and after the gluten challenge compared with administration of the placebo in patients with NCGS without adverse events. In particular, the changes in the score of the gluten-induced incomplete evacuation feeling and headaches were significantly improved. The serum levels of interleukin (IL)-8, tumor necrosis factor (TNF)-α, andregulated on activation, normal T cell expressed and secreted (RANTES) in subjects were not significantly changed by gluten, as expected from previous studies, and the enzyme mixture did not affect these inflammatory markers.

**Conclusion:**

In this human clinical study, we demonstrated the efficacy of the enzyme mixture derived from microorganisms and papaya in improving the symptoms of NCGS.

## Introduction

The population of individuals who experience gluten intolerance symptoms after the ingestion of food containing wheat gluten has increased^1^. The symptoms, which include fatigue, headache, and gastrointestinal distress (gas, bloating, diarrhea, constipation, vomiting, and reflux), reduce the quality of life of patients^2^ and reduce work productivity.

Gluten intolerance is classified into three major types of gluten-related disorders, which are autoimmune celiac disease (CD), non-celiac gluten sensitivity (NCGS), and wheat allergy^3–6^. CD is an autoimmune enteropathy caused by genetic and environmental factors, with an estimated worldwide prevalence of ~1%. Most individuals with gluten intolerance do not have CD but they experience improvement in symptoms when they limit their intake of gluten-containing food^7,8^. These patients are classified as having NCGS and considered to account for 6% of the US population^1^. Up to 15–30 million people currently have purchased and evaluated gluten-free food products that are currently readily available on the market. However, for most people, following a strict gluten-free diet is not practical because of the cost and the fact that many foods contain hidden gluten, on account of it being a common additive in the food industry.

Gluten is a protein found in wheat that is broken down into smaller fragments (peptides) and amino acids, which are the building blocks of proteins, by the human digestive system and then absorbed in the intestine as nutrients. However, the unusual peptide structure of one peptide fragment of gluten prevents it from being completely digested^3^. This fragment (33-mer, containing 33 amino acids LQLQPF[PQPQLPY]3PQPQPF) does not cause problems in most people but in patients with CD the 33-mer peptide causes a cascade of auto-immune reactions that severely damage the surface of the intestine^4^. Gliadin has also been suspected to be one of the triggers of NCGS^5,9^, because it increases intestinal permeability and innate immune cell activation^5^.

Recently multiple enzyme replacement therapy was suggested to be effective in patients with CD^10^ and gluten intolerance, including those with both CD and NCGS^11^. To clarify whether the symptoms of gluten intolerance, particularly in NCGS, could be improved by the administration of digestive enzymes, we designed an enzyme mixture, which can especially digest the 33-mer peptide efficiently. The 33-mer peptide contains six overlapping copies of three different DQ2-restricted epitopes and several preferable sites for tissue transglutaminase, which produce deamidated forms of gluten peptides that are more toxic than the amidated forms^12^. It is preferable to digest the 33-mer into particles of the smallest size possible. The 33-mer peptide contains a structure that is rich in proline, which is indigestible in the human digestive system. Therefore, we attempted to use digestive enzymes derived from microorganisms. For the mixture, we selected four enzymes with characteristics that suggest they might be able to digest the 33-mer peptide into small sizes.

Furthermore, it was also reported that gliadin does not induce mucosal inflammation or basophil activation in NCGS^13^. The mechanism of NCGS remains unclear. A clinical study of NCGS involving the removal of the 33-mer peptide from gluten in the digestive system would provide new knowledge related to the possibility of the 33-mer peptide being a trigger of NCGS.

In this human clinical study, we demonstrated the efficacy of our designed enzyme mixture in improving symptoms of NCGS.

## Materials and methods

### Materials

The enzyme mixture was a mixture of equal amounts of four enzymes. These enzymes were selected based on their potential ability to digest the 33-mer peptide efficiently. The selected enzymes had the following desirable characteristics. Peptidase derived from *Aspergillus oryzae* contains leucine aminopeptidase (Amano Enzyme Inc., Nagoya, Japan), which cleaves peptides carrying the N-terminal leucine at the highest rate^14^. The semi alkaline protease derived from *Aspergillus melleus* (Amano Enzyme Inc.) is an endopeptidase with high catalytic activity against hydrophobic amino acids residues^15–17^. Deuterolysin derived from *Penicillium citrinum* (Amano Enzyme Inc.) has a high affinity for P–X (X = G, K, L, or R)^18^ while cysteine protease derived from *Carica papaya* L. (EDC Inc., NY, USA) is non-specific endopeptidase that also efficiently digests gliadin^19^. Therefore, the enzyme mixture used in this study had characteristics that were compatible for digesting the 33-mer peptide to ≤6-mer peptides under simulated stomach condition in vitro. The prescription is described in Table 1 and dextrin was used as the placebo.

**Table 1:** The enzyme mixture and placebo prescriptions for one serving

Contents	Origin	Enzyme activity	Enzyme mixture	Placebo
Peptidase	*Aspergillus oryzae*	Peptidase activity, Amano LNA method, pH 7.0, 4260 µ/g	62.5 mg	–
Semi alkaline protease	*Aspergillus melleus*	Protease activity, Folin method, pH 8.0B, 1,330,000 µ/g	62.5 mg	–
Deuterolysin	*Penicillium citrinum*	Protease activity, FCC method, pH 7.0, 43,700 µ/g	62.5 mg	–
Cysteine protease	*Carica papaya*	Protease activity, common botanical protease assay methods, 1260 TU/mg	62.5 mg	–
Dextrin		–	–	250 mg
Total			250 mg	250 mg

### Subjects and procedure

**Fig. 1:**
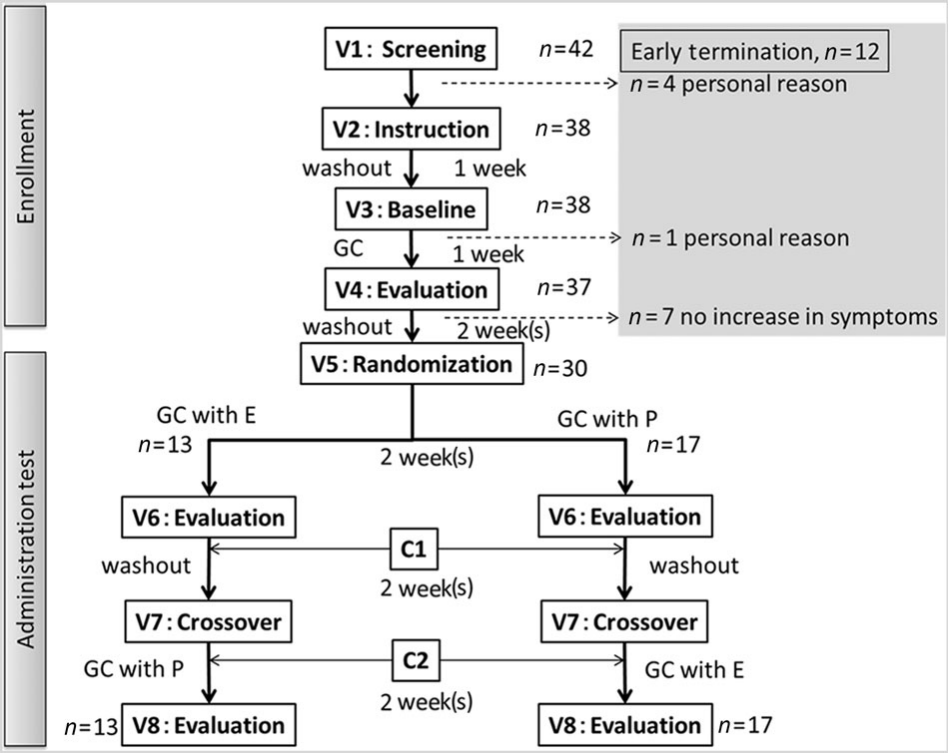
The schematic flowchart describing the procedure of the clinical research. V1–8, the subjects attended the visit following overnight fasting (at least 8 h) for data collection and commencement of the next steps. C1 and 2, phone check to determine the subjects’ compliance with the diet and assess any adverse events. GC gluten challenge, GC with E gluten challenge with the enzyme mixture, GC with P gluten challenge with placebo

A schematic flowchart describing the study procedure is shown in Fig. 1. The present study was designed as a randomized, single-blind, placebo-controlled crossover study and was conducted in the US. The visit 1 (V1) interview for screening potential participants was conducted. Male and female patients >18 years old who experienced gluten intolerance symptoms and the positive effect of a gluten-free or gluten-reduced diet, and have never been diagnosed with celiac disease, were recruited. Prior to performing any protocol-related procedures, the patients signed the Informed Consent and Health Insurance Portability and Accountability Act (HIPAA) Authorization forms with the approval of the Institutional Review Board (Liberty IRB, Inc., Deland, US; registration number: UMIN000028295). The safety was monitored by measuring vital signs, using a glucose diary, recording adverse events, and performing physical examination, standard hematology, urine　analysis, and electrocardiography (ECG). The subjects did not have chronic health conditions such as wheat allergy, type I or II diabetes, and cardiovascular disease (Table 2). Pregnant or lactating women were also excluded. Forty-two volunteers were screened as subjects with gluten intolerance symptoms by screening with a demographic interview, medical evaluation, and anti-gluten antibody test (Table 2. Furthermore, those who were strongly positive for both anti-transglutaminase and anti-gliadin levels were diagnosed as having CD and excluded from the study^20^). This clinical study was performed as exploratory research and involved the screening of an enforceable number of subjects with NCGS. The study procedure was as follows. V2: instruction and commencement of a gluten-free diet (washout) for 1 week. V3: the subjects responded to the celiac symptom questionnaire index (CSI, according to Leffeler et al.^2^) and started the gluten-challenge for 1 week. For the gluten challenge, the subjects were instructed to maintain a gluten-free diet with the addition of one slice of wheat bread (~1.5 g gluten) to each meal (three times per day, for a total of ~4.5 g gluten daily, the amount that causes symptoms of NCGS^20,21^). V4: subjects completed the CSI and started the 2-week washout. Individuals who do not experience a significant increase (≥30% of the V3 score) in clinical symptoms from V3 to V4 underwent early termination from the study. In this study, we designated subjects as patients with NCGS if they satisfied both conditions for non-diagnosis of CD in the antibody tests and showed a significant increase in the CSI score from V3 to V4. V5: the subjects were randomized according to a simple randomization method. One group was gluten-challenged with the enzyme mixture for 2 weeks, and the other was gluten-challenged with placebo for 2 weeks. They were administered two capsules containing 250 mg enzyme mixture or dextrin for placebo three times daily. The subjects’ compliance with the diet and dosing was determined during the phone checks (C1 and 2) and visits (V3–V8). V6: evaluation was performed, and the 2-week washout commenced. Furthermore, the subjects completed the CSI. V7: the crossover was initiated. V8: additional evaluation was performed and the study ended. The subjects completed the CSI and their health conditions were evaluated. The inflammatory factor levels of the subjects were analyzed at V3, V4, V6, V8, C1, and C2. Any individual who experienced significant inflammatory symptoms for 3 consecutive days during the subsequent 7- or 14-day gluten challenge advanced immediately to the next step at V3, C1, and C2.

**Table 2:** Study exclusion criteria

Criteria for excluding patients of other diseases	
Hemoglobin A1c	>10.0%
Hemoglobin	<10.0 g/dL
Congestive heart failure	Class III or IV
Blood pressure	>170/105
Liver function test	>2.5 (upper limits of normal)
Glomerular filtration rate	<30 mL/min
Criteria for excluding celiac disease	Possible CD diagnosis
Tissue transglutaminase antibody IgG, serum	≥6 U/mL
Tissue transglutaminase antibody IgA, serum	≥4 U/mL
Gliadin antibody IgG	≥20 U/mL
Gliadin antibody IgA	≥20 U/mL

Subjects who fulfilled any of the criteria described here were excluded from this study. Subjects diagnosed with other diseases or possible celiac disease were excluded

### Symptom questionnaire

The outcomes were measured based on the severity of symptoms using a symptom chart questionnaire with a qualitative scale utilizing a published and validated disease-specific index for adults with CD, the CSI^2^.

### Cytokine and chemokine assay

Blood samples were collected from the subjects and stored at −70 °C until all samples were ready for the analysis of interleukin (IL)-8, tumor necrosis factor (TNF)-α, and RANTES (regulated on activation, normal T cell expressed and secreted) using an enzyme-linked immunosorbent assay (ELISA) (Boster Biological Technology, CA, USA). All tests were performed according to the manufacturer’s instructions and the data were analyzed using the SoftMax Pro ver 5.4 (Molecular Devices, CA, USA).

### Statistical analysis

The data are expressed as the median, range, or mean ± standard error (SE). Statistical analysis was performed using the paired-*t* test. For all the tests, *p < 0.05 was considered significant.

## Results

### Enzyme mixture improved gluten-induced symptoms of NCGS

**Fig. 2:**
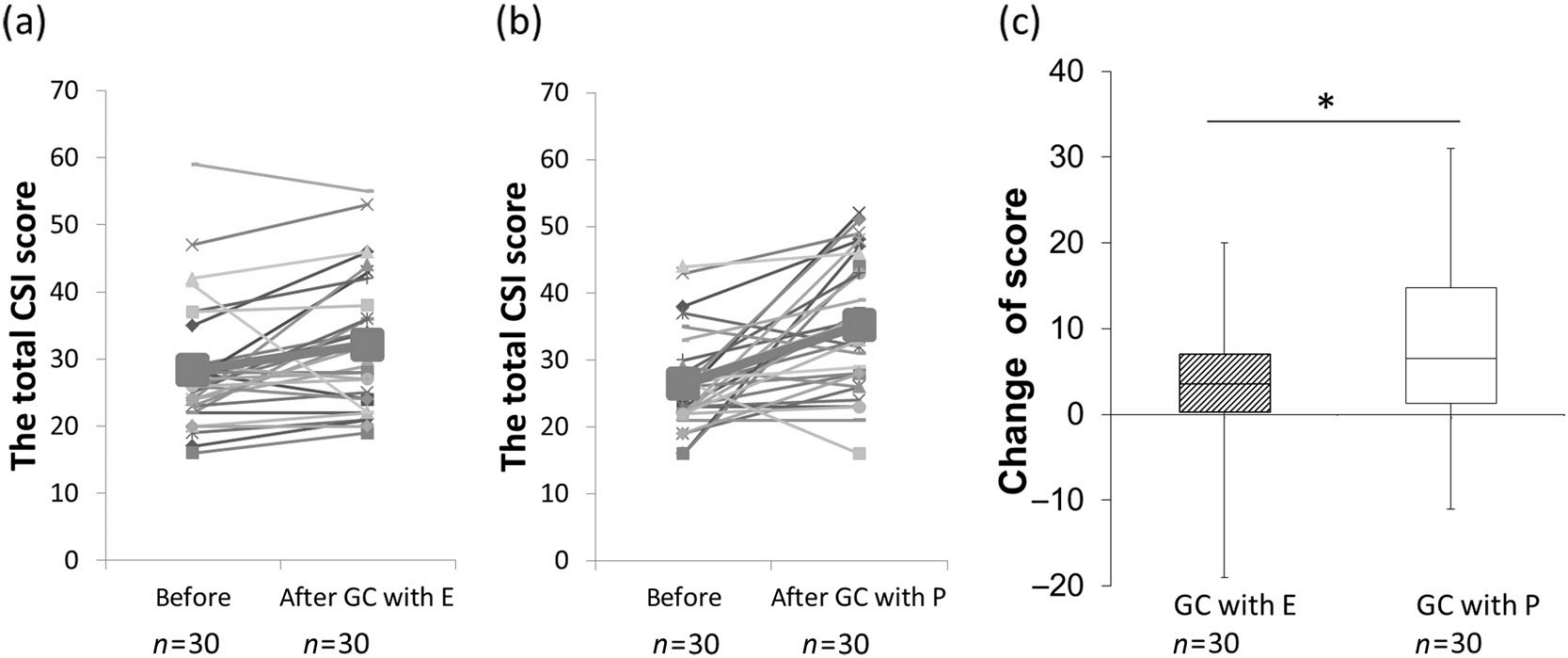
The effects of addministration of enzyme mixture or placebo to the change in total CSI score by gluten challenge. **a**, **b** The total CSI score before (Fig. 1, V5 or V7) and after (Fig. 1, V6 or V8) gluten challenge (GC) with enzyme mixture (E; (**a**)) or placebo (P; (**b**)). Thick lines and the largest symbols indicate average data. **c** The change in total CSI score from after washout with gluten-free diet for 2 weeks (Fig. 1, V5 or V7) to after gluten challenge with the enzyme mixture or dextrin as placebo for 2 weeks (Fig. 1, V6 or V8). Box with hatch marks indicates enzyme administration (GC with E, gluten challenge with enzyme mixture). White box indicates placebo (GC with P, gluten challenge with placebo). *p < 0.05

**Fig. 3:**
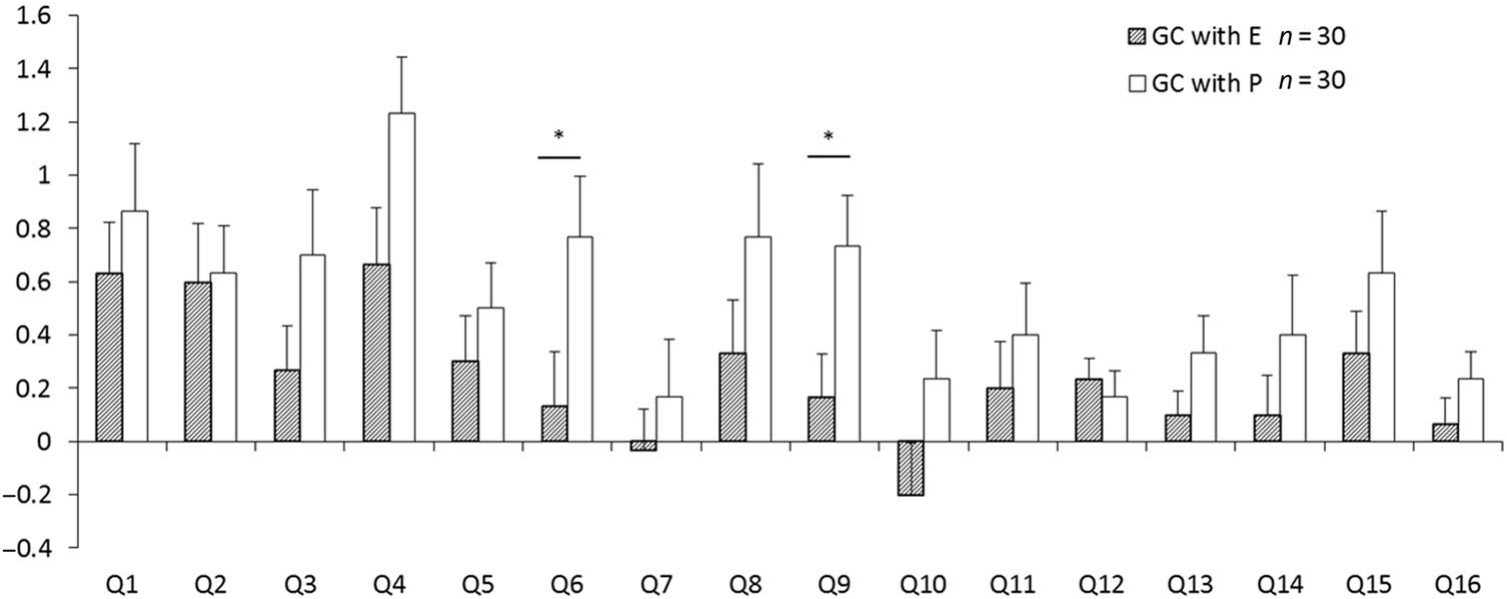
The changes in score of each question from after washout with gluten-free for 2 weeks (Fig. 1, V5 or V7) to after gluten challenge with the enzyme mixture or dextrin as placebo for 2 weeks (Fig. 1, V6 or V8). Questions are related to; Q1, pain or discomfort to the upper abdomen or the pit of the stomach. Q2, nausea. Q3, rumbling in your stomach. Q4, bloating. Q5, diarrhea. Q6, incomplete evacuation feeling. Q7, hunger pains. Q8, low energy level. Q9, headaches. Q10, food craving. Q11, loss of appetite. Q12, health condition related to CD. Q13, Overall health. Q14, physical pain. Q15, comfortable. Q16, healthy. Boxes with hatch marks indicate enzyme administration (GC with E). White boxes indicate placebo (GC with P). Data are expressed as mean ± SE. *p < 0.05

Thirty NCGS subjects (five male and 25 female subjects) completed the clinical tests and followed the diet and dosing. The subjects’ characteristics are described in Table 3. The total CSI scores before and after gluten challenge with enzyme mixture (a) or placebo (b) are shown in Fig. 2. The change in the total CSI score of the administration group from after washout (gluten-free for 2 weeks; Fig. 1, V5/V7) to after gluten challenge with the enzyme mixture for 2 weeks (Fig. 1, V6/8) was significantly lower than that of the placebo group (Fig. 2c). The results indicate that the symptoms of NCGS were improved by the enzyme mixture, since the total CSI score indicated the clinical status of the patients^2^. We also detected several improvements in the responses to each CSI question (Fig. 3). There were significant decreases in the scores of incomplete evacuation feeling (Q6) and headaches (Q9), and there was a decreasing trend in the score of bloating (Q4, *p* < 0.052) for those administered the enzyme mixture compared with those administered the placebo. However, no significant differences were observed for the other questions. The enzyme mixture could be expected to improve representative symptoms for patients with NCGS, especially bloating, incomplete evacuation feeling, and headaches. There was no significant difference in values of the measured items listed in Table 3 between the enzyme administration and placebo groups at V8 (weight, body mass index [BMI], and blood components). No adverse events induced by the enzyme mixture were detected during the safety evaluation or based on the scores of each question in the CSI (Fig. 3).

**Table 3:** Subject characteristics

Age	41.5, 22–74
Height (cm)	165, 151–199
Weight (kg)	83.6, 59.0–154.2
BMI	30.6, 22.0–54.7
Anti-transglutaminase antibody IgA (U/mL)	<1.5
Anti-transglutaminase antibody IgG (U/mL)	<2.3
Anti-gliadin antibody IgA (U/mL)	5, 2–14
Anti-gliadin antibody IgG (U/mL)	2, <1.0–3
CHL (mg/dL)	182, 131–269
HDL (mg/dL)	59.5, 25.5–109.4
TRIG (mg/dL)	106, 44–211
LDL (mg/dL)	103, 30–187
HA1c (%)	5.2, 4.6–9.2
CRP (mg/dL)	2.4, <0.2–14.4

*BMI* body mass index, *CHL* cholesterol, *HDL* high-density lipoprotein, *TRIG* triglycerides, *LDL* low-density lipoprotein, *HA1c* hemoglobin A1c, *CRP* C-reactive protein

Data are expressed as median and range

**Fig. 4:**
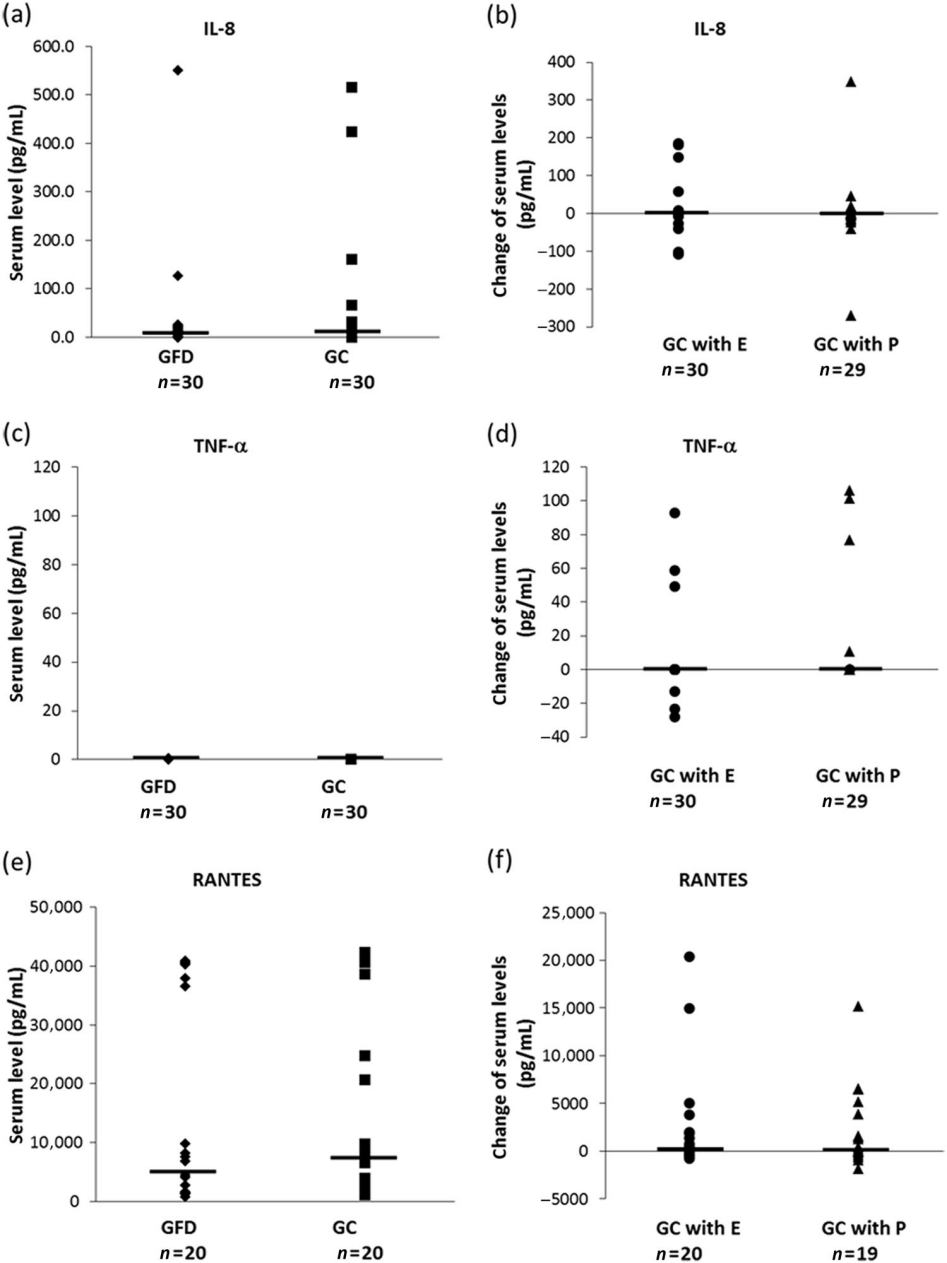
The serum levels of inflammatory markers during this clinical study. The serum levels of IL-8, TNF-α, and RANTES under the condition of the gluten-free diet (Fig. 1, V3) or gluten challenge (Fig. 1, V4) are shown in **a**, **c**, and **e**. The changes in serum levels of IL-8, TNF-α, and RANTES from after washout with gluten-free (Figure1, V3, V5 or V7) to after gluten challenge, gluten challenge with the enzyme mixture, or gluten challenge with dextrin as placebo (Figure1, V4, V6 or V8) are shown in **b**, **d**, and **f**. No significant difference was detected in each component by statistical analysis performed by paired *t*-test. ◆GFD gluten free diet, ■GC gluten challenge, ●GC with E gluten challenge with the enzyme mixture, ▲GC with P gluten challenge with placebo. Horizontal bars are median values

For further discrimination of NCGS, we measured the serum levels of IL-8 and TNF-α because these inflammatory markers do not increase in NCGS^5,21^ but they do in CD^22,23^. As a candidate inflammatory marker of NCGS, the serum levels of RANTES, produced in dendritic cells upon gliadin stimulation^22^, were also measured. In this study, we did not detect significant differences in the serum levels of IL-8, TNF-α, or RANTES following consumption of gluten, gluten with the enzyme mixture, or gluten with the placebo (Fig. 4). We were unable to collect a blood sample from one subject at V6 and, therefore, the n of gluten challenge with placebo (GC with P) is less than that of gluten challenge with enzyme mixture (GC with E; Fig. 4b, d, f). The RANTES data of several subjects whose blood sample measurements were out of range of the serum level of RANTES even in a single measurement were excluded (Fig. 4e, f). These results are consistent with those of a previous study of NCGS and indicate that the enzyme mixture did not affect the serum levels of these innate cytokines and chemokines.

## Discussion

In this study, we recruited patients with NCGS and showed that their symptoms were improved by administration of the enzyme mixture without adverse events within the observed range. Several studies of enzyme replacement therapy for gluten intolerance have been reported for CD and gluten intolerance including both CD and NCGS^10,11^. However, in this present study, we excluded patients with CD using a demographic interview, medical evaluation, anti-gluten antibody test, and gluten challenge test. The symptoms of both CD and NCGS are thought to be caused by the indigestible 33-mer peptide, which is generated during the digestion of gliadin in the digestive system and induces mucosal inflammation^3–6^. Recently, however, it was considered that the mechanism of NCGS cannot be explained by the 33-mer peptide only^12,13,24^. We tested whether the symptoms of NCGS were improved by digesting the 33-mer peptide in the digestive system using the digestive enzymes derived from microorganisms and papaya. The digestive enzymes enabled the digestion of this indigestible protein in the human digestive system. We used CSI score to evaluate effects on NCGS symptoms according to the methods of Lffeler et al.^2^ Lffeler and colleagues showed that a CSI score of 30 or less was associated with high QOL and excellent GDF adherence, and that a score of 45 or greater was associated with relatively poor QOL and ongoing active celiac disease. The extent to which the enzyme mixture decreased the change in score upon gluten challenge in this report is considered to be clinically meaningful. Therefore, this study showed that the administration of the enzyme mixture improved the symptoms of patients with NCGS. This finding indicates that the symptoms of NCGS are not induced in the absence of the 33-mer peptide in the digestive system even when patients with NCGS consumed gluten.

Gliadin induces the release of IL-8 and TNF-α from dendritic cells by crossing the epithelial barrier in patients with CD^21^. Serum levels of IL-8 and TNF-α are higher in patients with active CD than in healthy adult controls^23^. However, the levels of released IL-8 and TNF-α from ex vivo-cultured duodenal biopsies of patients with NCGS following a gluten consumption did not increase, although that of IL-8 from patients with CD increased^21^. Consistent with this previous report, serum levels of the inflammatory markers IL-8 and TNF-α were not increased upon gluten challenge in patients with NCGS in this study. These results confirmed that the recruited subjects had NCGS.

RANTES may be a chemokine marker of CD because gliadin triggers the release of RANTES from dendritic cells^22^. However, levels of RANTES in the supernatants of ex vivo-cultured duodenal biopsies from NCGS and CD patients were not changed by gliadin^21^. We showed that the serum levels of RANTES were not changed upon gluten challenge in patients with NCGS. This suggests that RANTES cannot be an inflammatory marker of NCGS. Although the baseline serum levels of IL-8, TNF-α, and RANTES exhibited large individual differences, gluten induces increases in the serum levels of IL-8 and TNF-α in patients with CD^23,25^. Changes in the serum levels of IL-8 and TNF-α after GC with E and GC with P were not significantly large compared with those of previous reports. RANTES is known as an inflammatory marker, as serum levels of RANTES increase under inflammatory conditions in patients with unstable angina pectoris or idiopathic retroperitoneal fibrosis^26,27^. Changes in the serum levels of RANTES by GC, GC with E, and GC with P were also not significant compared with those of previous reports. These results indicate that administration of the enzyme mixture did not affect the serum levels of these inflammatory markers but improved the symptoms of NCGS.

Recently, gliadin was considered not to trigger symptoms of NCGS because only half of the patients with NCGS with the HLA-DQ2 or DQ8 haplotype, or both, showed induction of mucosal inflammation by gliadin^12,13^. Several components in wheat are considered as candidates for the triggering factors, e.g., gluten-related proteins, amylase-trypsin inhibitors, fermentable oligosaccharides, disaccharides, and polyols^24,28,29^. However, the factors and mechanisms of NCGS are still unclear. Our results suggest that the enzyme mixture, which digested the 33-mer peptide, can resolve the triggering factors of NCGS possibly including the 33-mer peptide. The one-dose enzyme mixture digested almost all 33-mer peptide from a meal under the conditions of the stomach at pH 4.5 in vitro (data not shown). Furthermore, the enzyme mixture digested almost all the proteins in wheat under conditions that mimicked the stomach environment after a meal in vitro (data not shown). The improvement of symptoms of NCGS induced by gluten could be mediated by the digestion of gluten-related or other wheat proteins by our enzyme mixture. Because the distinct factors that cause NCGS are unclear, we considered the possibilities that multiple factors could induce NCGS and that multiple types of NCGS exist. However, our results indicate that the symptoms of not but just some types of patients of NCGS could be improved by our enzyme mixture.

## Conclusions

This clinical research demonstrated that our enzyme mixture, which was composed of peptidases and protease derived from microorganisms and papaya, improved the symptoms of patients with NCGS. Our enzyme mixture was designed to digest the 33-mer peptide generated from gliadin and could be expected to be a useful dietary supplement to decrease the symptoms of NCGS in patients without adverse events within the observation period.

## Study Highlights

### What is current knowledge

- The population of individuals with non-celiac gluten sensitivity (NCGS) has increased.
- Gluten-related proteins have been considered triggers of NCGS.
- Therefore, enzyme replacement therapy is expected to be effective.

### What is new here

- We designed a new enzyme mixture containing peptidase, semi alkaline protease, deuterolysin, and cysteine protease.
- We screened patients with NCGS and performed a clinical trial using a gluten challenge with the enzyme mixture.
- The new enzyme mixture of protease and peptidase efficiently improved the symptoms of NCGS.
